# Effect of Polymer Phase Transition Behavior on Temperature-Responsive Polymer-Modified Liposomes for siRNA Transfection

**DOI:** 10.3390/ijms20020430

**Published:** 2019-01-19

**Authors:** Kenichi Nagase, Momoko Hasegawa, Eri Ayano, Yoshie Maitani, Hideko Kanazawa

**Affiliations:** Faculty of Pharmacy, Keio University, 1-5-30 Shibakoen, Minato, Tokyo 105-8512, Japan; peach-hasegawa.3720048@z6.keio.jp (M.H.); ayano-er@pha.keio.ac.jp (E.A.); maitani-y@u01.gate01.com (Y.M.)

**Keywords:** thermoresponsive polymer, siRNA delivery, liposome, transfection

## Abstract

Small interfering RNAs (siRNAs) have been attracting significant attention owing to their gene silencing properties, which can be utilized to treat intractable diseases. In this study, two temperature-responsive liposomal siRNA carriers were prepared by modifying liposomes with different polymers—poly(*N*-isopropylacrylamide-*co*-*N*,*N*-dimethylaminopropyl acrylamide) (P(NIPAAm-*co*-DMAPAAm)) and poly(*N*-isopropylacrylamide-*co*-*N*,*N*-dimethylacrylamide) P(NIPAAm-*co*-DMAAm). The phase transition of P(NIPAAm-*co*-DMAPAAm) was sharper than that of P(NIPAAm-*co*-DMAAm), which is attributed to the lower co-monomer content. The temperature dependent fixed aqueous layer thickness (FALT) of the prepared liposomes indicated that modifying liposomes with P(NIPAAm-*co*-DMAPAAm) led to a significant change in the thickness of the fixed aqueous monolayer between 37 °C and 42 °C; while P(NIPAAm-*co*-DMAAm) modification led to FALT changes over a broader temperature range. The temperature-responsive liposomes exhibited cellular uptake at 42 °C, but were not taken up by cells at 37 °C. This is likely because the thermoresponsive hydrophilic/hydrophobic changes at the liposome surface induced temperature-responsive cellular uptake. Additionally, siRNA transfection of cells for the prevention of luciferase and vascular endothelial growth factor (VEGF) expression was modulated by external temperature changes. P(NIPAAm-*co*-DMAPAAm) modified liposomes in particular exhibited effective siRNA transfection properties with low cytotoxicity compared with P(NIPAAm-*co*-DMAAm) modified analogues. These results indicated that the prepared temperature-responsive liposomes could be used as effective siRNA carriers whose transfection properties can be modulated by temperature.

## 1. Introduction

Nucleic acid therapeutics have been investigated as potential treatments for intractable diseases [1]. One promising group of nucleic acid therapeutics is small interfering RNAs (siRNAs) because they induce messenger RNA (mRNA) degradation in cells and RNA interference, leading to the suppression of gene expression [1,2,3,4]. Various diseases caused by the expression of pathogenic protein can therefore be treated with siRNA.

However, it is challenging to effectively deliver siRNA to cells because of the electrostatic repulsion between the siRNA and the cell membrane, and the instability of siRNA. To overcome these challenges, siRNA carriers have been investigated to facilitate delivery into cells. Cationic liposomes are widely used as nucleic acid carriers since the cationic charge of the liposomes leads to the formation of a complex with the anionic nucleic acid fragments—called a lipoplex—which enhances cellular uptake. However, cationic liposomes are unstable in vivo because they tend to aggregate with serum proteins. Therefore, polymer coated liposomes have been investigated. Polyethylene glycol (PEG) modification of liposomes leads to excellent stability in the blood stream [5,6,7]. However, PEG modified liposomes exhibit low cell interaction as a result of their hydrophilic properties, resulting in lower delivery efficiency. Therefore, temperature-responsive polymer-modified liposomes have been investigated to achieve temperature modulated cellular uptake [8,9,10,11]. Poly(*N*-isopropylacrylamide) (PNIPAAm) is often used as a temperature responsive polymer for liposome modification.

PNIPAAm is widely used as a thermoresponsive polymer in biomedical applications such as drug delivery [12,13,14], bioseparation [15,16,17,18,19], biosensing, bioimaging and diagnostic devices [20,21,22], and cell culture substrates for tissue engineering and regenerative medicines [23,24,25,26,27,28,29,30]. PNIPAAm exhibits temperature-responsive hydrophilic and hydrophobic changes across its lower critical solution temperature (LCST) of 32 °C, which can be attributed to hydration and dehydration of the polymer chain [31,32,33], and thermoresponsive polymer-modified liposomes have been developed based on this property. Below the LCST, PNIPAAm modified liposomes are hydrophilic, which prevents cell uptake. In contrast, above the LCST, temperature-modulated liposomes become hydrophobic, leading to enhanced cellular uptake. However, the intrinsic LCST of PNIPAAm is 32 °C, which is not appropriate for use at physiological temperature (37 °C). To tune the LCST of the polymer to ~37 °C, copolymerization of hydrophilic monomer into the PNIPAAm chain has been reported [34,35,36]. *N*,*N*-Dimethylacrylamide (DMAAm) and *N*,*N*-dimethylaminopropyl acrylamide (DMAPAAm) have frequently been used as co-monomers for modulation of the LCST of PNIPAAm to body temperature [37,38,39,40,41]. Both DMAAm and DMAPAAm are acrylamide monomers with relatively hydrophilic properties compared with PNIPAAm, and their introduction into the polymer chain results in the elevation of the LCST. DMAPAAm has cationic properties and is therefore strongly hydrophilic. Therefore, a relatively low degree of copolymerization of DMAPAAm into PNIPAAm is sufficient for LCST modulation [40,41]. In contrast, DMAAm is neutral compared with DMAPAAm and exhibits relatively weak hydrophilic properties. Therefore, a larger amount of DMAAm is required in the copolymerization for modulation of the LCST [37,38,39]. If these polymers were used for liposome modification to produce siRNA carriers, the difference in the polymer properties would affect the temperature responsive behavior of the modified liposome carriers, leading to efficient siRNA transfection.

**Figure 1:**
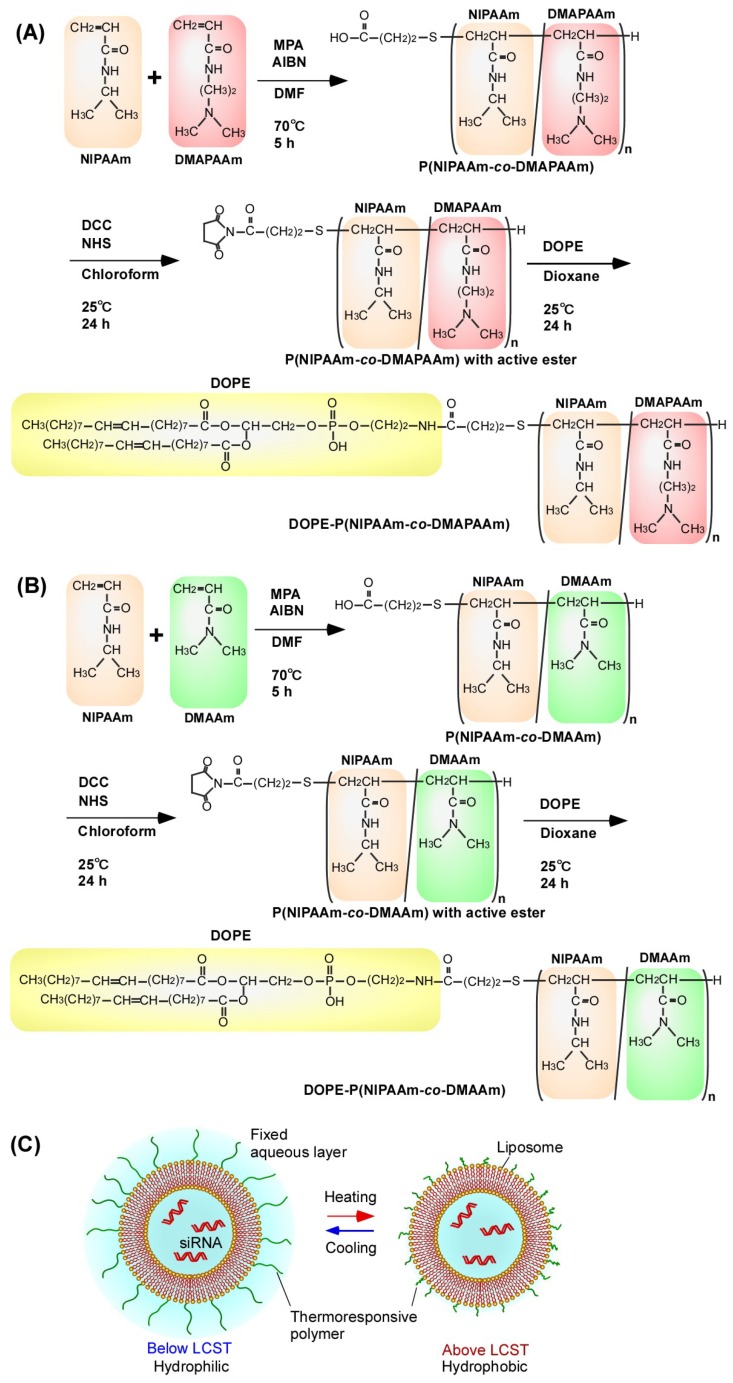
Schemes for the preparation of (**A**) P(NIPAAm-*co*-DMAPAAm) modified DOPE and (**B**) P(NIPAAm-*co*-DMAAm) modified DOPE. (**C**) Illustration of a thermoresponsive polymer-modified liposome as an siRNA carrier.

In this study, two types of thermoresponsive polymer-modified liposome were prepared using P(NIPAAm-*co*-DMAPAAm) and P(NIPAAm-*co*-DMAAm) (Figure 1). Characterization of the liposomes was carried out, including evaluation of the temperature-responsive property changes. Additionally, temperature-modulated siRNA transfection for suppression of luciferase and VEGF expressions in cells was performed. The polymer most appropriate for liposome modification was determined from the findings.

## 2. Results and Discussion

### 2.1. Characterization of the Prepared Polymers

**Figure 2:**
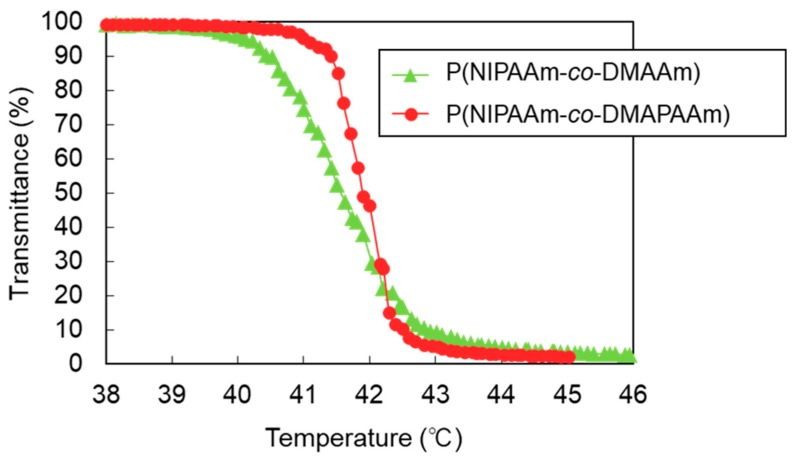
Phase transition behavior of the thermoresponsive polymers in phosphate buffer saline (PBS). The lower critical solution temperature (LCST) was defined as the temperature at which the optical transmittance of the polymer solution was 50% of the difference between the maximum and minimum transmittances.

The properties of the synthesized polymers were investigated using gel permeation chromatography (GPC), ^1^H NMR, and titration (Table 1). The phase transition profiles of the prepared polymers were also measured (Figure 2). GPC showed that the molecular weights of P(NIPAAm-*co*-DMAPAAm) and P(NIPAAm-*co*-DMAAm) were 17,000 and 15,000 g/mol, respectively. The molecular weight of the polymers determined by GPC was higher than expected as the polymerization procedure was designed to give polymers with a molecular weight of 10,000 g/mol. It is possible that the determined molecular weights were inaccurate because the calibration of the GPC columns was performed using polyethylene glycol standards, therefore, the molecular weights were also determined by ^1^H NMR and acid-base titration. We experimentally assessed whether polymer molecular weight determination using ^1^H NMR or acid-base titration is more accurate than using GPC in this case. The molecular weight of P(NIPAAm-*co*-DMAAm) was determined by acid-base titration because titration is known to be a relatively accurate method for measuring molecular weight compared with using ^1^H NMR or GPC. However, it is difficult to determine the molecular weight of P(NIPAAm-*co*-DMAPAAm) by acid-base titration because P(NIPAAm-*co*-DMAPAAm) is a weak base. We therefore measured the molecular weight of P(NIPAAm-*co*-DMAPAAm) by ^1^H NMR, despite molecular weight observation using ^1^H NMR being less accurate than titration.

As a result, it was concluded that the molecular weights of P(NIPAAm-*co*-DMAPAAm) and P(NIPAAm-*co*-DMAAm) were 11,000 and 13,500, respectively. We consider these values to be more accurate than those determined by GPC.

The phase transition behavior of the prepared polymers was established from the temperature-dependent transmittance change of the solution. The LCSTs of P(NIPAAm-*co*-DMAPAAm) and P(NIPAAm-*co*-DMAAm) were observed at 41.7 °C and 41.9 °C, respectively, which are appropriate temperatures for temperature-modulated cellular uptake of liposomes. In addition, P(NIPAAm-*co*-DMAPAAm) exhibited a sharp phase transition profile, while the phase transition of P(NIPAAm-*co*-DMAAm) proceeded over a broad temperature range. This difference is attributed to the copolymer content. P(NIPAAm-*co*-DMAPAAm) consisted of 94 mol % NIPAAm and 6 mol % DMAPAAm, while P(NIPAAm-*co*-DMAAm) consisted of 69.5 mol % NIPAAm and 30.5 mol % DMAAm. Previous reports have indicated that the phase transition of PNIPAAm copolymer developed a broad profile as co-monomer content increased [42,43]. Therefore, P(NIPAAm-*co*-DMAPAAm) with low co-monomer content, exhibited a sharp transition profile.

### 2.2. Characterization of the Prepared Liposomes

The prepared liposomes were characterized by measuring their size, polydispersity index (PDI), and zeta potential (Table 2). The size of the non-modified liposomes was 135.8 nm. The polymer-modified liposomes exhibited a slightly larger size than the unmodified liposomes, indicating that the modified polymer extended to the outer surface of the liposomes. All liposomes exhibited small PDI values indicating that the procedure used in this study resulted in uniformly sized liposomes. Zeta potential measurement of the non-modified liposomes showed a large value, which is attributed to the cationic properties of 1,2-dioleoyl-3-trimethylammonium propane (chloride salt) (DOTAP). The polymer-modified liposomes exhibited lower zeta potential values than the unmodified liposomes. This is because the presence of the polymer on the liposome shields the cationic properties of DOTAP. Additionally, after the liposome-siRNA complex formation, the zeta potential value decreased. This is because the cationic properties of DOTAP were reduced by complex formation with siRNA.

**Figure 3:**
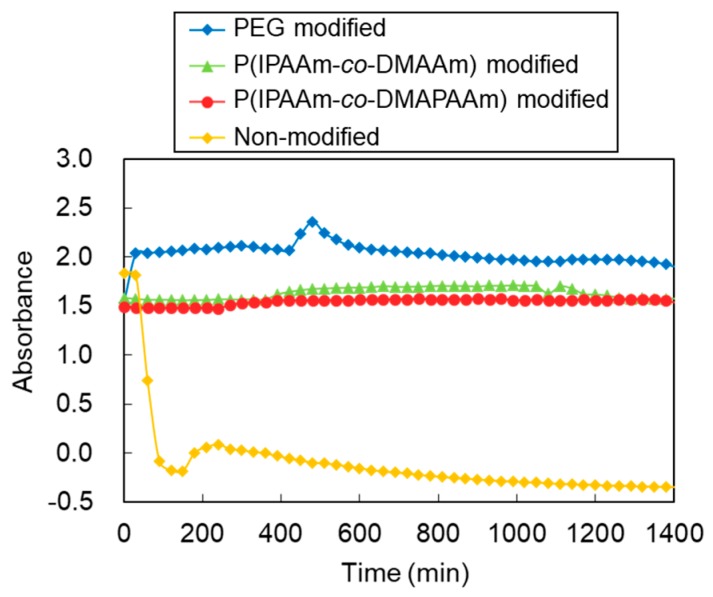
Stability of liposomes in serum for 24 h at 37 °C. Monitoring wavelength: 450 nm. Serum concentration: 50 *v*/*v*%.

The stability of the liposomes was investigated by measuring the time course of the absorbance of the liposome suspension with human serum (Figure 3). When the liposomes were suspended in serum solution, the suspension exhibited absorbance. In contrast, when liposomes formed aggregates with serum proteins, they tended to settle out of suspension leaving a clear supernatant and reducing the absorbance. The absorbance of the non-modified liposome suspension decreased promptly, indicating that non-modified liposomes tend to aggregate with human serum. In contrast, for liposomes modified with PEG, P(NIPAAm-*co*-DMAPAAm), and P(NIPAAm-*co*-DMAAm), the absorbance of the suspension was maintained. This result indicates that polymer-modified liposomes have high stability even in the presence of serum.

**Figure 4:**
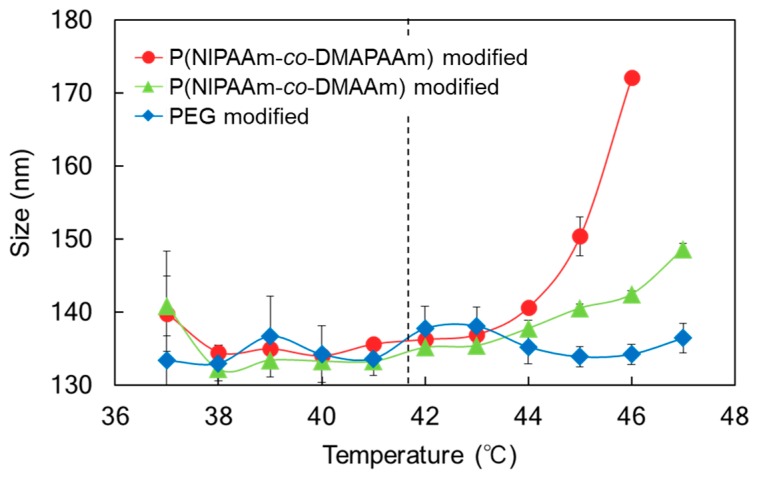
Temperature dependent size change of liposomes in PBS. The dashed line indicates the LCST of the thermoresponsive polymers.

The temperature-dependent size change of the liposomes was observed using PBS as the suspension solvent (Figure 4). PEG modified liposomes maintained their size as the temperature was increased. In contrast, P(NIPAAm-*co*-DMAPAAm) and P(NIPAAm-*co*-DMAAm) modified liposomes exhibited an increase in size above the LCST of the polymers. At lower temperature, P(NIPAAm-*co*-DMAPAAm) and P(NIPAAm-*co*-DMAAm) were hydrated and extended, leading to the formation of an aqueous outer liposome layer, which prevented the aggregation of the liposomes. Above the LCST, the polymers became hydrophobic, which is attributed to dehydration, leading to a reduction in the aqueous layer of the liposomes. The reduced aqueous layer induced aggregation of the liposomes, leading to an increase of the observed liposome size. In addition, above the LCST of the polymers, P(NIPAAm-*co*-DMAPAAm) modified liposomes exhibited a clear increase in size compared with P(NIPAAm-*co*-DMAAm) modified liposomes. One possible reason for this observation is the difference in the conformations of P(NIPAAm-*co*-DMAPAAm) and P(NIPAAm-*co*-DMAAm) following shrinkage. Above the LCST, both polymers change their conformation to the shrunken state. P(NIPAAm-*co*-DMAPAAm) has a slight positive charge in the polymer chain, leading to suppression of shrinkage. Therefore, the strong positive charge of the liposome was shielded by the polymer above the LCST, leading to aggregation of the liposomes. In contrast, P(NIPAAm-*co*-DMAAm) has no positive charge and tends to be compact and shrunken above LCST. Therefore, the strong positive charge of the liposome was exposed, leading to suppression of the aggregation of the liposomes owing to electrostatic repulsion.

**Figure 5:**
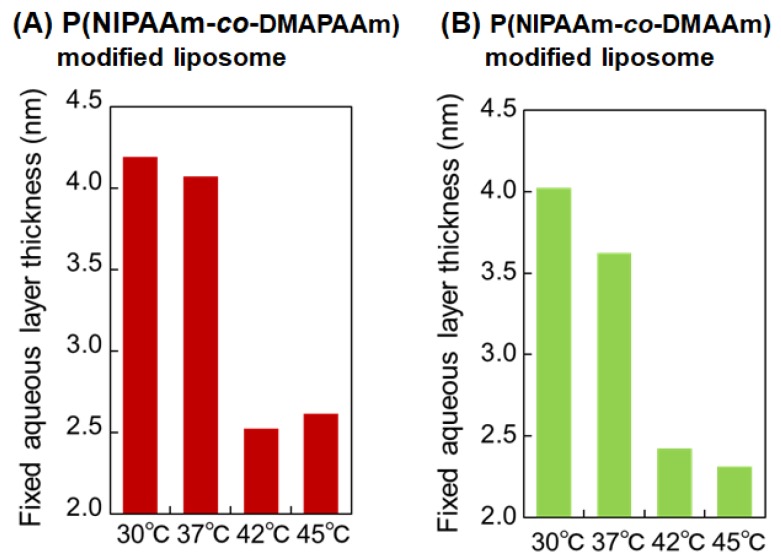
Temperature dependence of the fixed aqueous layer thickness (FALT) of temperature-responsive liposomes. The FALT was calculated using Gouy-Chapman theory.

To investigate the aqueous layer of the thermoresponsive polymer-modified liposomes, the fixed aqueous layer thickness was measured at various temperatures using a previously reported procedure [10] (Figure 5). Both of the thermoresponsive polymer-modified liposomes exhibited reduced aqueous layer thickness with increasing temperature. P(NIPAAm-*co*-DMAPAAm) modified liposomes in particular exhibited notable aqueous layer reduction across the LCST, while the aqueous layer of P(NIPAAm-*co*-DMAAm) gradually decreased over a broad temperature range from 30 °C to 42 °C. This observation is attributed to the difference in the phase transition properties between P(NIPAAm-*co*-DMAPAAm) and P(NIPAAm-*co*-DMAAm). Since P(NIPAAm-*co*-DMAAm) has a large DMAAm content (30.5 mol %), a broad phase transition proceeded with increasing temperature. In contrast, P(NIPAAm-*co*-DMAPAAm) contained a low DMAPAAm composition (6 mol %) and exhibited a sharp phase transition temperature. The fixed aqueous layer of P(NIPAAm-*co*-DMAPAAm) liposomes therefore exhibited a marked decrease across the LCST.

### 2.3. Cellular Uptake of Liposomes

**Figure 6:**
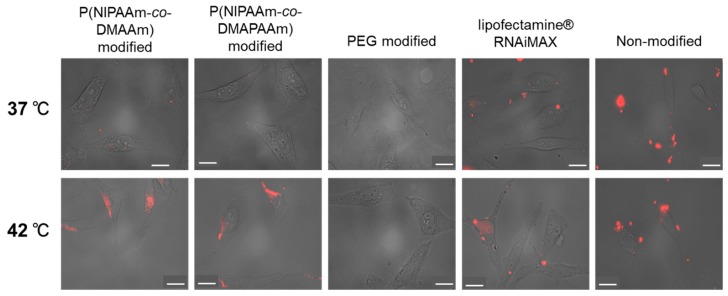
Cellular uptake of liposome-siRNA complex at 37 °C and 42 °C using Alexa fluor 555 labeled siRNA and luciferase expressing HeLa cells. The liposome-siRNA complex and cells were incubated for 4 h at 37 °C and 42 °C. The images were prepared by merging the phase contrast cell images and fluorescent siRNA images. The scale bar indicates 20 μm.

To investigate the temperature-dependent cellular uptake properties of the liposome-siRNA complex, cellular uptake was observed at 37 °C and 42 °C using Alexa fluor 555 labeled siRNA and fluorescence microscopy (Figure 6). Non-modified liposomes tended to aggregate, leading to an uneven distribution outside the cells. PEG modified liposomes were not taken up at 37 °C or 42 °C because the liposomes were relatively hydrophilic. Lipofectamine RNAiMax, a commercially available transfection reagent, was effectively taken up into cells at both 37 °C and 42 °C, and an even distribution of Alexa fluor 555 labeled siRNA was observed in cells. In the case of the temperature-responsive liposomes, cellular uptake of the liposome-siRNA complex was observed at 42 °C, but was not observed at 37 °C. This observation is attributed to the temperature-responsive properties of the polymer on the liposome surface. The P(NIPAAm-*co*-DMAPAAm) and P(NIPAAm-*co*-DMAAm) on the liposome surface became hydrophobic at 42 °C, leading to an enhanced hydrophobic interaction between the cells and liposomes. Therefore, the liposome-siRNA complex was taken up into cells at 42 °C. In contrast, thermoresponsive polymer was hydrophilic at 37 °C, leading to prevention of temperature responsive polymer-modified liposome uptake. In addition, a previous investigation reported that the cellular uptake of temperature-responsive liposomes occurred via microtubule-dependent transport and clathrin-mediated endocytosis rather than caveolin-mediated endocytosis [10,11]. These results indicate that the prepared thermoresponsive polymer-modified liposomes can modulate siRNA transfection through simple temperature change.

### 2.4. Gene Silencing Activity of the Prepared Liposomes

**Figure 7:**
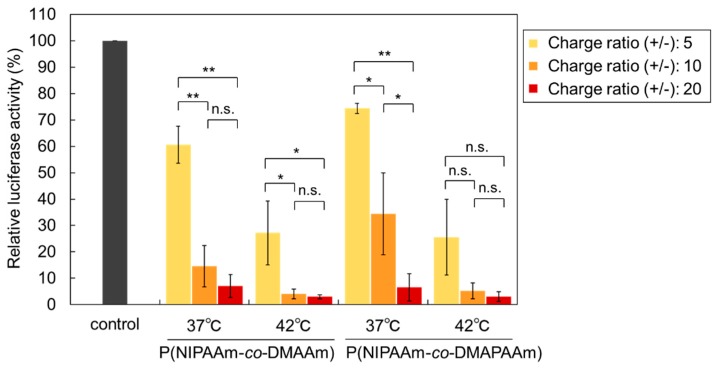
Effect of the charge ratio on the gene silencing activity of Luc-HeLa cells transfected with Luc-siRNA. siRNA was transfected for 4 h at 37 °C or 42 °C using liposomes and lipofectamine RNAiMAX. The data are mean ± standard deviation (SD) (*n* = 3 or 4, ** *p* < 0.01, * *p* < 0.05, and n.s.: not significant).

In order to investigate the gene silencing activity of the prepared temperature responsive liposomes, liposome-siRNA complexes were prepared and siRNA transfection was evaluated using luciferase expressing HeLa cells. First, the optimal charge ratio (+/−) of the prepared thermoresponsive liposomes was investigated (Figure 7). Luciferase activity was reduced by increasing the charge ratio (+/−) because electrostatic interaction between the liposome and cell membrane increased with increasing charge ratio (+/−). However, cytotoxicity is expected to increase with increasing charge ratio (+/−). In addition, at high charge ratio (+/−), luciferase activity was high even at 37 °C, indicating that temperature modulation was not effective. In contrast, at a charge ratio (+/−) of 5:1, temperature-modulated gene silencing was observed. Relatively low gene silencing activity was observed at 37 °C, and high gene silencing was observed at 42 °C. The results show that a charge ratio (+/−) of 5:1 provides optimal conditions for temperature-modulated transfection of siRNA.

**Figure 8:**
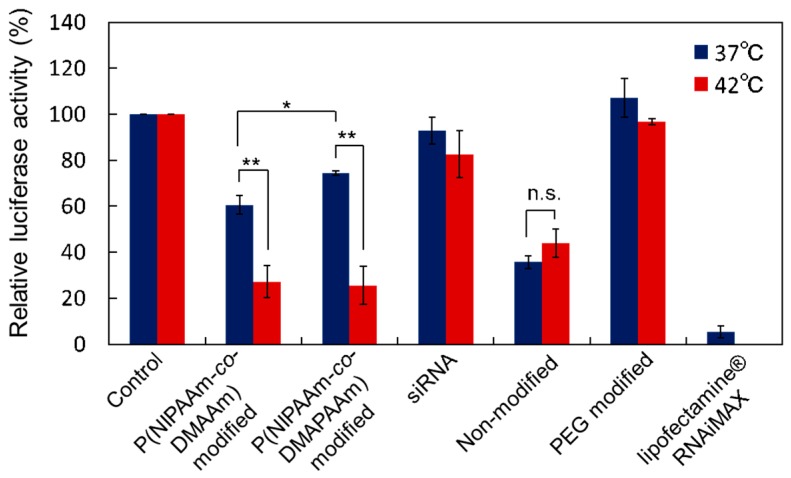
Effect of temperature on gene silencing activity of Luc-HeLa cells transfected with Luc-siRNA. siRNA was transfected for 4 h at 37 °C or 42 °C using liposomes and lipofectamine RNAiMAX. The charge ratio (+/−) was 5:1 and the siRNA concentration was 25 nM. The data are mean ± standard deviation (SD). (*n* = 3 or 4, ** *p* < 0.01, * *p* < 0.05, and n.s.: not significant).

With a charge ratio (+/−) of 5:1, temperature-dependent gene silencing activity was observed using temperature-responsive liposomes (Figure 8). Non-modified liposomes, PEG modified liposomes, commercially available transfection reagent, and siRNA alone were also measured for comparison. When only siRNA was used for transfection, luciferase activity was not reduced at either 37 °C or 42 °C, indicating that siRNA was not taken up by the cells. PEG modified liposomes did not reduce the luciferase activity at either 37 °C or 42 °C because the PEG modified liposomes did not tend to be taken up owing to their hydrophilic properties. Non-modified liposomes reduced the luciferase activity at both temperatures, indicating that the liposome-siRNA complex was taken up by the cells and siRNA was delivered to suppress the luciferase activity. Both temperature-responsive liposomes reduced luciferase activity at 42 °C, but it was not suppressed at 37 °C. This result indicates that temperature-responsive liposomes can modulate siRNA delivery into cells and perform gene silencing as a result of external temperature change. This is because the thermoresponsive polymer on the liposomes becomes hydrophobic above the LCST, resulting in liposome uptake and siRNA delivery into the cells. In contrast, below the LCST, the thermoresponsive polymers become hydrophilic, leading to prevention of cellular uptake. In addition, P(NIPAAm-*co*-DMAPAAm) modified liposomes exhibited more effective temperature-modulated siRNA delivery into cells compared with the P(NIPAAm-*co*-DMAAm) modified liposome. This is because P(NIPAAm-*co*-DMAPAAm) has sharp phase transition properties compared with those of P(NIPAAm-*co*-DMAAm), which is attributed to the lower co-monomer content of P(NIPAAm-*co*-DMAPAAm).

**Figure 9:**
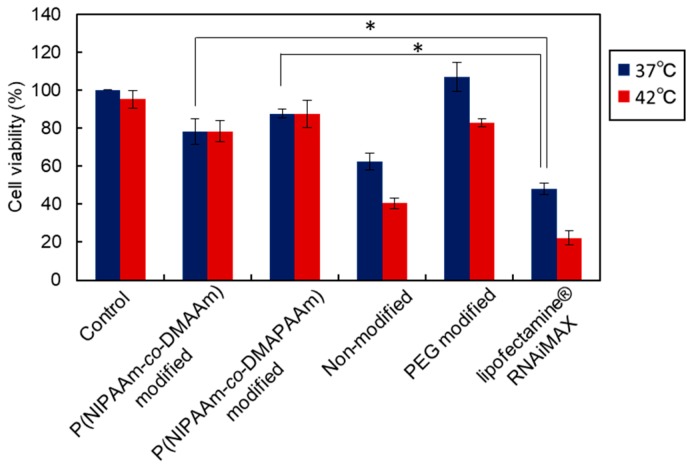
Cell viability of Luc-HeLa cells. siRNA was transfected for 4 h at 37 °C or 42 °C followed by a 20 h incubation at 37 °C. Cell viability was evaluated by WST-8 assay. The data are mean ± standard deviation (SD) (*n* = 3 or 4, * *p* < 0.05).

Cell viability after the siRNA transfection was evaluated by WST-8 assay (Figure 9). Non-modified liposome and Lipofectamine RNAiMax reduced the cell viability, which is likely a result of the cationic properties of the non-modified liposome and Lipofectamine RNAiMax. In contrast, thermoresponsive liposomes exhibited high cell viability compared with the non-modified liposome and Lipofectamine RNAiMax. This is likely because the modified polymer on the liposome surface suppressed the cytotoxicity of the cationic liposome. In addition, P(NIPAAm-*co*-DMAPAAm) modified liposome exhibited a slightly higher cell viability compared with P(NIPAAm-*co*-DMAAm) modified liposome. This result indicates that P(NIPAAm-*co*-DMAPAAm) modified liposome has a high transfection ability as well as low cytotoxicity. Therefore, P(NIPAAm-*co*-DMAPAAm) modified liposome is an appropriate temperature-responsive siRNA carrier.

**Figure 10:**
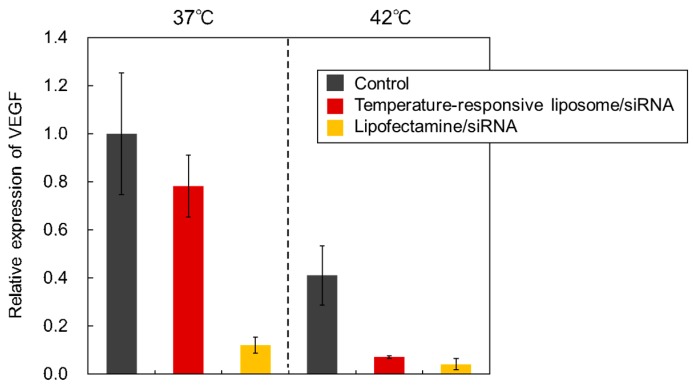
VEGF gene silencing using liposome-siRNA complexes in HeLa cells. Cells were treated with complexes for 4 h at 37 °C or 42 °C followed by incubation for 44 h at 37 °C. VEGF mRNA levels were estimated by real time-PCR normalized to β-actin mRNA. The relative expression of VEGF was estimated by the VEGF expression level of HeLa cells at 37 °C as a control. The error bars indicate the range of the relative expression of VEGF using real time PCR. The dotted line indicates the difference in incubation temperature.

Suppression of the VEGF expression of HeLa cells was carried out using P(NIPAAm-*co*-DMAPAAm) modified liposomes (Figure 10). If VEGF expression of cancer cells in vivo is suppressed, angiogenesis and cancer cell growth can be prevented, leading to the possibility of using liposome-siRNA complexes as anti-cancer therapeutics. VEGF expression was estimated from the mRNA of VEGF. The VEGF expression was suppressed at 42 °C compared with the 37 °C case. Previous reports have indicated that the VEGF expression of tumor cells was suppressed by heating at hyperthermia [44]. Therefore, the suppressed VEGF expression at 42 °C was reasonable. Using temperature-responsive liposomes as siRNA carriers for silencing VEGF expression effectively suppressed VEGF expression at 42 °C, which is the same as was found when Lipofectamine RNAiMax was used as the siRNA carrier. This result indicated that temperature-responsive liposomes can modulate VEGF expression of cancer cells with temperature change, leading to the possibility of suppressing VEGF expression of cancer cells in vivo, thereby inhibiting cancer cell growth.

These results indicated that P(NIPAAm-*co*-DMAPAAm) modified liposomes would be useful as siRNA carriers whose transfection can be modulated by external temperature. In addition, the temperature-responsive liposomes would be applicable not only in cell transfection in vitro, but also cancer therapy with hyperthermia.

## 3. Materials and Methods

### 3.1. Materials

*N*-isopropylacrylamide (NIPAAm), *N*,*N*-dimethylacrylamide (DMAAm), and *N*,*N*-dimethylaminopropylacrylamide (DMAPAAm) were kindly provided by KJ Chemicals (Tokyo, Japan). NIPAAm was recrystallized from *n*-hexane. DMAAm and DMAPAAm were distilled. 2,2-azobisisobutyronitrile (AIBN), *N*,*N*-dimethylformamide (DMF), diethylether, acetone, bromothymol blue (BTB), L-α-phosphatidyl ethanolamine, dioleoyl (DOPE), and chloroform were obtained from Wako Pure Chemical Industries, Ltd. (Osaka, Japan). Methanol, 3-mercaptopropionic acid (MPA) and *N*,*N*′-dicyclohexylcarbodiimide (DCC) were obtained from Kanto Chemical (Tokyo, Japan). Chloroform-d (CDCl_3_) was obtained from Tokyo Chemical Industries (Tokyo, Japan). 1,2-dioleoyl-3-trimethylammonium propane (chloride salt) (DOTAP) and 1,2-distearoyl-*sn*-glycero-3-phosphoethanolamine-*N*-[amino(polyethylene-glycol)-2000] (ammonium salt) (DSPE-PEG2000) were obtained from Avanti Polar Lipids (Alabaster, AL, USA). Dulbecco’s phosphate-buffered saline (D-PBS), Dulbecco’s modified eagle medium (DMEM), Non-Essential Amino Acid Solution (NEAA), and minimum essential media (MEM) were purchased from Thermo Fisher Scientific (Waltham, MA, USA). BLOCK-iTTM Alexa Fluor^®^ Red Fluorescent Control siRNA and lipofectamine RNAiMAX transfection reagent were also purchased from Thermo Fisher Scientific (Waltham, MA, USA). HeLa cells stably expressing a firefly luciferase (HeLa-Luc) were donated by Dr. Kenji Yamato (Tsukuba University, Tsukuba, Japan). HeLa cells were obtained from RIKEN cell bank (Tsukuba, Japan).

siRNAs were obtained from Sigma Aldrich (St Louis, MO, USA). The sequences were as follows: suppression of luciferase: sense: 5′-CCGUGGUGUUCGUGUCUAATT-3′; antisense: 5′-UUAGACACGAACACCACGGTT-3′, suppression of VEGF: sense: 5′-GGAGUACCCUGAUGAGAUCTT-3′; antisense: 5′-GAUCUCAUCAGGGUACUCCTT-3′.

### 3.2. Synthesis of Thermoresponsive Copolymers

Two types of thermoresponsive copolymer—P(NIPAAm-*co*-DMAPAAm) and P(NIPAAm-*co*-DMAAm)—were synthesized by radical polymerization using MPA as a chain transfer agent (Figure 1).

In the synthesis of P(NIPAAm-*co*-DMAAm), NIPAAm (1.87 g, 16.5 mmol) and DMAAm (1.13 g, 7.2 mmol) (a NIPAAm to DMAAm ratio of 69.5:30.5), were dissolved in 6 mL of DMF in a flask. Then, MPA (31.8 mg, 0.30 mmol) and AIBN (9.8 mg, 0.06 mmol) were added to the solution. The reaction solution was deoxygenated by argon gas bubbling for 20 min, and the flask was purged with nitrogen. The flask was then sealed, and the polymerization was allowed to proceed at 70 °C for 5 h. After the polymerization, the copolymer was purified by reprecipitation using diethyl ether (300 mL) twice.

For P(NIPAAm-*co*-DMAPAAm) synthesis, NIPAAm (2.76 g, 24.36 mmol) and DMAPAAm (243.2 mg, 1.56 mmol) (a NIPAAm to DMAPAAm ratio of 94.0:6.0), were polymerized using the procedure described for P(NIPAAm-*co*-DMAAm).

### 3.3. Characterization of Synthesized Polymers

The phase transition behavior of the prepared polymers was observed by temperature dependent transmittance change of the polymer solution. Polymer solution was prepared by dissolving the polymer in PBS at a concentration of 5 mg/mL. The transmittance of the polymer solution at 500 nm was measured using a UV-Vis spectrometer (V-630, JASCO, Tokyo, Japan) whilst heating the polymer solution at 0.1 °C/min. The lower critical solution temperature was defined as the temperature at which 50% transmittance was observed.

The molecular weight of the polymer was obtained by GPC (GPC-8020, Tosoh, Tokyo, Japan) using two serially connected TSK-Gel α-M columns (Tosoh, Tokyo, Japan). The columns were calibrated using PEG standards. The mobile phase was DMF containing 10 mM of LiCl. The molecular weight of P(NIPAAm-*co*-DMAPAAm) was also determined by ^1^H NMR. The sample solution was prepared by dissolving copolymers in CDCl_3_ at a concentration of 15 mg/mL. ^1^H NMR spectra were obtained using a nuclear magnetic resonance spectrometer (Varian INOVA 500, Varian, Palo Alto, CA, USA). The molecular weight of P(NIPAAm-*co*-DMAAm) was also obtained by titration molecular weight. The polymer (25 mg) was dissolved in 2 mL of pure water. Titration of the terminal carboxyl group of the polymer was performed using 0.0025 mol/L NaOH solution.

### 3.4. Conjugation of Thermoresponsive Polymer to Lipid

To modify the liposomes with thermoresponsive polymer, the polymer was conjugated to the lipid component, L-α-phosphatidylethanolamine, dioleoyl (DOPE). P(NIPAAm-*co*-DMAAm) (544 mg, 0.0403 mmol), DOPE (30 mg, 0.0403 mmol), NHS (20.8 mg, 0.1008 mmol), and DCC (11.6 mg, 0.101 mmol) were dissolved in 4 mL of chloroform in a flask. The flask was then purged with nitrogen and the reaction was allowed to proceed for 24 h. After the reaction, the solvent was evaporated, and methanol was added. The solution was dialyzed using dialysis membrane of MWCO 3500 (Spectrum Laboratories, Rancho Dominguez, CA, U.S.A) for 2 days. After the dialysis, the solution was evaporated, and the sample was dried in vacuo. The obtained thermoresponsive polymer conjugated lipid was stored at −30 °C. In the case of P(NIPAAm-*co*-DMAPAAm), P(NIPAAm-*co*-DMAPAAm) (443 mg, 0.0403 mmol) was used, and conjugation of the polymer to DOPE was carried out using the same reaction. The conjugation of the thermoresponsive polymers to DOPE was confirmed by ^1^H NMR using CDCl_3_ as the solvent.

### 3.5. Preparation of Thermoresponsive Polymer-Modified Liposomes

Liposomes were prepared using DOTAP and DOPE because DOTAP has cationic properties and tends to form a complex with anionic siRNA, and DOPE has fusogenic properties for interaction with the cell membrane, leading to enhanced cellular uptake. Four types of liposome were prepared using the following procedure: DOTAP, DOPE, and P(NIPAAm-*co*-DMAAm)-DOPE were added to a flask at a molar ratio of 3:6.5:0.5, respectively, and dissolved in 3 mL of chloroform. The chloroform was then evaporated and a lipid film was formed on the internal surface of the flask. Pure water (1 mL) was added to the lipid film, which was then dispersed using a vortex mixer. The dispersed solution was added to a test tube and sonicated for 30 min. The prepared liposome suspension was then extruded using an extruder with 100 nm pore diameter (Avanti Polar Lipids, Alabaster, AL, USA) to give P(NIPAAm-*co*-DMAAm) modified liposomes.

P(NIPAAm-*co*-DMAAm) modified liposomes and PEG modified liposomes were prepared in the same manner using P(NIPAAm-*co*-DMAPAAm)-DOPE or DSPE-PEG2000 in place of P(NIPAAm-*co*-DMAAm)-DOPE. To prepare non-modified liposomes, DOTAP and DOPE were used at the molar ratio of 3:7, respectively.

To prepare liposomes complexed with siRNA (lipoplexes), each liposome suspension (20 mg total lipid/mL water) and siRNA aqueous solution (200 mM stock concentration) were separately diluted with cell culture medium, mixed at a charge ratio (+/−) of 5:1 by vortex-mixing for 10 s, and left for 30 min at room temperature. The liposome-siRNA complexes, with a final concentration of 50 nM siRNA, were used immediately after the preparation. The theoretical charge ratio (+/−) of cationic liposome to siRNA was calculated as the molar ratio of DOTAP to siRNA phosphate. Lipofectamine RNAiMAX (RNAiMAX) (Invitrogen, Corp., Carlsbad, CA, USA) was used for comparison, and transfection procedures were performed in accordance with the manufacturer’s instructions.

### 3.6. Liposome Characterization

The size of the prepared liposomes was measured by dynamic light scattering. The liposome suspension was diluted 40 times with pure water or PBS, and the sample suspension was measured using a Zetasizer Nano-ZS (Malvern Instruments, Malvern, UK) to give the mean diameter of the liposomes.

The zeta potential of the liposomes was measured with an electrophoretic light scattering apparatus, ELSZK-2 KOP apparatus (Otuska Electronics, Osaka, Japan). The prepared liposomes were diluted 50 times with pure water. Zeta potential was then measured at 25 °C.

The stability of the prepared liposomes was evaluated by incubation of the liposomes with human serum. Commercially available human serum was diluted with 3 mL of pure water. The serum solution and the prepared liposome suspension was mixed at a ratio of 1:1. The absorbance of the liposome suspension was monitored at 450 nm for 24 h.

The fixed aqueous layer thickness (FALT) of the prepared liposome was measured using previously reported methods [10]. The liposomes were prepared using a 9 weight percent sucrose solution. Sucrose solutions (9 wt %) with various NaCl concentrations (0, 5, 10, and 20 mmol/L) were prepared. The prepared liposomes were diluted 50 times with sucrose solution containing various concentrations of NaCl. The zeta potential of the liposomes was measured at 30, 37, 42, and 50 °C. The FALT was calculated using the following equation with Gouy-Chapman theory [45,46]. According to this theory, zeta potential ζ(*L*) was calculated from the electrostatic potential at the position of the slipping plane *L* (nm) and was expressed as the following equation.

ln ζ(*L*) = ln*A* − *κL* (1)

where *A* is a constant; *κ* is the Debye-Hȕckel parameter—which is equal to √C/0.3, *C* is the molar concentration of electrolyte—and *L* is the slipping plane and FALT in nanometer.

### 3.7. Cell Culture

HeLa cells were cultured using MEM containing 10% fetal bovine serum (FBS), 1% MEM Non-Essential Amino Acids Solution (MEM NEAA), and 100 µg/mL penicillin streptomycin as the cell culture medium at 37 °C in 5% CO_2_. For passage culture, 0.05 wt/v% trypsin-EDTA solution was used for harvesting cells, and the cell suspension was reseeded onto a 75 cm^2^ cell culture flask and incubated for 3 or 4 days.

Luciferase expressing HeLa cells (HeLa-Luc) were cultured using high glucose DMEM containing 10% fetal bovine serum (FBS), 1% MEM NEAA, and 100 μg/mL G-418 sulfate as the cell culture medium at 37 °C in 5% CO_2_. For passage culture, 0.05 weight/volume% trypsin-EDTA solution was used for harvesting cells and the cell suspension was reseeded onto a 75 cm^2^ cell culture flask and incubated for 3 or 4 days.

### 3.8. Gene Silencing of Luciferase Using siRNA Loaded Liposomes

The gene silencing capabilities of the prepared siRNA loaded liposomes were investigated using luciferase expressing HeLa cells (HeLa-Luc). HeLa-Luc cells were seeded in a 6-well plate at a density of 7.5 × 10^4^ cells/well. Cells were incubated at 37 °C in 5% CO_2_ for 24 h, and cells adhered to the wells. Liposome-siRNA complex was prepared at an siRNA concentration of 25 nM. The liposome-siRNA complex solution (1 mL) was added to the HeLa-Luc incubated wells and a subsequent incubation was performed for 4 h at 37 °C or 42 °C. The cells were then rinsed with 1 mmol/L EDTA in PBS to remove liposomes adsorbed on the cell surfaces, and subsequently incubated at 37 °C for 44 h. After the incubation, cells were rinsed with PBS. Cells were then lysed to evaluate the luciferase activity. Cell lysis buffer (Promega, Madison, WI, USA) was used by diluting the reagent five times with PBS, and 250 μL of diluted solution was added to the cells in the wells. The samples were then incubated at 37 °C for 30 min, after which the cell suspension was removed using a pipette and transferred to a 1 mL tube. The tube was incubated at −80 °C for 10 min to completely lyse the cells. The suspension was then centrifuged at 12,000 rpm for 5 min. The supernatant (10 μL) was collected and 50 μL of luciferase assay reagent (PicaGene^®^ Luminescence Kit, Tokyo Ink, Tokyo, Japan), was added to the solution. The luminescence intensity was observed using a microplate reader (TECAN Infinite M1000, Zürich, Switzerland). In addition, the protein concentration of the 10 μL of lysate was measured using a BCA protein assay kit (Thermo Fisher Scientific, Waltham, MA, USA). Luciferase activity in the cell lysates was measured as counts per second (cps), and the obtained value was divided per amount of protein (g) to cancel out the error due to differences in the amount of cells. Luciferase activity (%) was obtained from the ratio of transfected cells to untreated cells.

### 3.9. Suppression of VEGF Using siRNA Loaded Liposomes

Suppression of VEGF expression was carried out using temperature responsive liposomes containing siRNA. Lipoplexs were prepared using VEGF suppressing siRNA [47,48]. The sequence is shown in the supporting information. HeLa cells were seeded in a 6-well plate at a density of 5.0 × 10^4^ cells/well. Cells were incubated at 37 °C in 5% CO_2_ for 24 h, and cells adhered to the well. Liposome-siRNA complex was prepared at an siRNA concentration of 25 nM. The Liposome-siRNA complex solution (1 mL) was added to the HeLa cells in the well, which were subsequently incubated for 4 h at 37 °C or 42 °C. The cells were then rinsed with 1 mmol/L EDTA in PBS to remove liposomes adsorbed on the cell surface, and a subsequent incubation was carried out at 37 °C for 44 h. An RNA extraction reagent (1 mL) (ISOGEN, Nippon Gene, Tokyo, Japan) was added to the cells, which were then incubated for 5 min at room temperature. The cell lysate suspension was transferred to a 1.5 mL tube, 200 μL of chloroform was added and the mixture was agitated with a vortex mixer. The suspension was centrifuged at 12,000 rpm for 15 min at 4 °C. The water layer (400 μL) was combined with 320 μL of isopropanol and mixed gently. The suspension was centrifuged at 13,800 rpm for 15 min at 4 °C, and the supernatant was added to 1 mL of ethanol. The solvents were then allowed to evaporate on a clean bench. RNase-free water (44 μL), DNase I (10 units), and DNase I buffer (5 μL) were added to the dried sample and incubated for 15 min. Then, RNase-free water (150 μL) and phenol-chloroform solution (150 μL) were added and the mixture was agitated with a vortex mixer. The solution was centrifuged at 12,000 rpm for 15 min at 4 °C and the water layer (150 μL) was collected. Sodium acetate solution (3 mol/L, pH 5.2, 15 μL) and ethanol (375 μL) were added to the collected aqueous layer solution. The solution was then incubated at −20 °C overnight. The solution was centrifuged at 15,000 rpm for 15 min at 4 °C and rinsed with 75% ethanol. The ethanol was removed by decanting, and the sample was dried on a clean bench for 1 h. The sample was then dissolved in 30 μL of RNase-free water, and the RNA concentration was measured. To adjust the RNA concentration to 0.05 μg/μL, 5.8 μL of Master Mix (Thermo Fisher Scientific, Waltham, MA, USA) and RNase-free water were added to the RNA solution. cDNA was synthesized by reverse transcription with a thermal cycler (C1000^TM^ Thermal Cycler, Bio-Rad, Hercules, CA, USA) at 25 °C for 10 min, 37 °C for 2 h, and 85 °C for 5 min.

SYBR Mix (Thermo Fisher Scientific, Waltham, MA, USA) (5 μL), forward primer (0.1 μL), reverse primer (0.1 μL), and RNase-free water (4.3 μL) were then added to the 200 μL tube. The synthesized cDNA (5 μL) was added to the mixed solution. PCR analysis was performed using a PCR apparatus with CFX Manager™ Software (Bio-Rad, Hercules, CA, USA). β-actin was used for normalization. The ∆∆Ct method was used for calculation. The PCR primer sequences used were as follows. VEGF forward primer: AGGAGGGCAGAATCATCACG; VEGF reverse primer: CAAGGCCCACAGGGATTTTCT; β-actin forward primer: GTGGGGCGCCCCAGGCACCAGGGC; β-actin reverse primer: CTCCTTAATGTCACGCACGATTTC.

### 3.10. Determination of Cellular Uptake by Fluorescence Microscopy

HeLa-Luc cells were seeded in a 35 mm glass bottom dish at a density of 5.0 × 10^4^ cells/well. Cells were incubated at 37 °C in 5% CO_2_ for 24 h, and cells adhered to the dish. Liposome-siRNA complex was prepared at an siRNA concentration of 30 nM and a charge ratio (+/−) of 5:1. Liposome and Alexa fluor 555 labeled siRNA were suspended in DMEM without FBS (Biosera, Boussens, France), and the suspension was incubated for 30 min. The cell culture medium in the well was then removed using an aspirator and the prepared liposome-siRNA complex solutions were added to the cells. The samples were then incubated at 37 °C and 42 °C for 4 h. The cells were rinsed with PBS containing 1 mmol/L EDTA. DMEM containing FBS was added to the cells and observation was carried out using a fluorescence microscope (Biorevo BZ-9000, Keyence, Osaka, Japan). The images were prepared by merging the phase contrast cell images and fluorescent siRNA images.

### 3.11. Cell Viability Assessment

The cytotoxicity of the prepared liposome-siRNA complex was evaluated by WST-8 assay. A HeLa cell suspension (5.0 × 10^4^ cells/mL, 100 µL) was seeded into a 96-well cell culture plate and incubated at 37 °C for 24 h. The liposome-siRNA complex was prepared at an siRNA concentration of 25 nM. The cell culture medium in the 96-well plate was then removed and the prepared siRNA-liposome complex solution was added to the cells in the 96-well plate. The incubation was performed at 37 °C or 42 °C for 4 h. After the incubation, the cells were rinsed with PBS containing 1 mM EDTA, 100 µL of cell culture medium was added, and the cells were incubated at 37 °C for 20 h. After the incubation, 10 µL of WST-8 reagent solution (Dojindo, Kumamoto, Japan) was added to the cells in the well, and further incubation was carried out at 37 °C for 1 h. The absorbance at 450 nm was then measured using a plate reader (TECAN Infinite M1000, Zürich, Switzerland).

### 3.12. Statistical Analysis

Student’s t-test was used for the statistical analysis of the obtained results. *p* < 0.05 was considered statistically significant.

## 4. Conclusions

Temperature-responsive liposomes were prepared by modifying liposomes with two types of thermoresponsive polymer—P(NIPAAm-*co*-DMAPAAm) or P(NIPAAm-*co*-DMAAm). siRNA transfection properties were investigated by comparing non-modified liposome, PEG modified liposome, and commercially available siRNA carrier. The phase transition behavior of P(NIPAAm-*co*-DMAPAAm) showed a sharp phase transition compared with that of P(NIPAAm-*co*-DMAAm) because P(NIPAAm-*co*-DMAPAAm) had a lower co-monomer content compared with P(NIPAAm-*co*-DMAAm). Additionally, P(NIPAAm-*co*-DMAPAAm) modified liposomes exhibited a large change in the thickness of the fixed aqueous monolayer between 37 °C and 42 °C, while that of P(NIPAAm-*co*-DMAAm) modified liposomes changed over a broad temperature range. The thermoresponsive polymer-modified liposomes exhibited high stability in serum solution at 37 °C, as did PEG modified liposomes, while the non-modified liposomes aggregated rapidly. The temperature-responsive liposomes exhibited cellular uptake at 42 °C, but were not taken up into cells at 37 °C. This is thought to be because the thermoresponsive hydrophilic/hydrophobic change of the liposome surface induced temperature responsive cellular uptake. Additionally, siRNA transfection of cells for prevention of luciferase activity and VEGF expression could be modulated by external temperature change. P(NIPAAm-*co*-DMAPAAm) modified liposomes in particular exhibited effective siRNA transfection properties with low cytotoxicity compared with P(NIPAAm-*co*-DMAAm) modified liposomes. These results indicated that the prepared temperature-responsive liposomes would be an effective siRNA carrier, whose transfection properties could be modulated by temperature.

## Figures and Tables

**Table 1:** Characterization of thermoresponsive polymers.

Polymer	Molecular Weight				LCST (°C) ^d^
	GPC		^1^H NMR ^b^	Titration ^c^	
	Mn ^a^	Mw ^a^			
P(NIPAAm-*co*-DMAPAAm)	17,000	4.4	11,000		41.9
P(NIPAAm-*co*-DMAAm)	15,000	3.5		13,500	41.7

^a^ Determined by GPC using polyethylene glycol as a standard and DMF containing 10 mM LiCl as the mobile phase, ^b^ Determined by ^1^H NMR using CDCl_3_ as a solvent. ^c^ Determined by titration of the terminal carboxyl group, ^d^ Defined as the temperature at which the optical transmittance of the polymer solution was 50% of the difference between the maximum and minimum transmittances.

**Table 2:** Characterization of the prepared liposomes.

Modified Polymer	Sample ^a^	Diameter ^b^ (nm)	PDI ^b^	Zeta Potential ^d^ (mV)
P(NIPAAm-*co*-DMAPAAm)	siRNA (−)	166.6 ± 1.5	0.14	43.05 ± 1.08
	charge ratio (+/−) = 5	168.3 ± 1.0	0.08	20.95 ± 0.13
	charge ratio (+/−) = 10	171.4 ± 7.9	0.07	27.78 ± 1.64
	charge ratio (+/−) = 20	169.1 ± 7.3	0.09	35.24 ± 0.36
P(NIPAAm-*co*-DMAAm)	siRNA (−)	164.2 ± 7.3	0.19	52.32 ± 1.39
	charge ratio (+/−) = 5	170.5 ± 2.4	0.12	23.28 ± 0.69
	charge ratio (+/−) = 10	158.1 ± 6.9	0.12	35.22 ± 0.72
	charge ratio (+/−) = 20	150.3 ± 0.4	0.13	38.79 ± 0.88
Non-modified	siRNA (−)	135.8 ± 0.7	0.24	62.06 ± 0.35
	charge ratio (+/−) = 5	N.D. ^c^	N.D. ^c^	29.16 ± 5.20
	charge ratio (+/−) = 10			47.45 ± 0.57
	charge ratio (+/−) = 20			50.67 ± 1.03

^a^ Charge ratio (+/−) was obtained from the molar ratio of DOTAP to siRNA phosphate. ^b^ Determined by measuring dynamic light scattering. ^c^ N.D. indicates not detected owing to precipitation. ^d^ Determined using a zeta potential analyzer.

## Abbreviations

AIBN	2,2-Azobisisobutyronitrile
BTB	Bromothymol blue
DCC	*N*,*N*′-Dicyclohexylcarbodiimide
DMAAm	*N*,*N*-Dimethylacrylamide
DMAPAAm	*N*,*N*-Dimethylaminopropyl acrylamide
DMEM	Dulbecco’s modified eagle medium
DMF	*N*,*N*-dimethylformamide
DOTAP	1,2-Dioleoyl-3-trimethylammonium propane
DOPE	L-α-phosphatidyl ethanolamine, dioleoyl
SPE	L-α-distearoyl-phosphatidylethanolamine
FALT	Fixed aqueous layer thickness
GPC	Gel permeation chromatography
LCST	Lower critical solution temperature
MEM	Minimum essential media
NEAA	Non-Essential Amino Acid Solution
NIPAAm	*N*-isopropylacrylamide
NMR	Nuclear magnetic resonance
PEG	Polyethylene glycol
siRNA	Small interfering RNA
VEGF	Vascular endothelial growth factor
